# Heart rate and EEG gamma band connectivity in the ventral attention network during emotional movie stimulation in women with high emotion dysregulation

**DOI:** 10.3389/fnins.2025.1599349

**Published:** 2025-06-25

**Authors:** Francesca Fusina, Marco Marino, Alessandro Angrilli

**Affiliations:** Department of General Psychology, University of Padova, Padua, Italy

**Keywords:** cardiovascular activity, emotion, brain connectivity, heart-brain connection, women, EEG

## Abstract

**Introduction:**

The heart-brain connection represents an interesting innovative framework for investigating the complex and reciprocal influences between the cardiovascular system and brain activity in emotion research. The present study aimed at investigating the correlation between heart rate and connectivity within brain regions relevant for interoception and emotional regulation (i.e., the Ventral Attention Network) during ecological stimulation with validated emotional video-clips.

**Methods:**

To this end two groups of 25 healthy female students were enrolled (mean age 22.62 ± 1.87SD), after a selection from 422 students, based on questionnaires measuring emotion dysregulation. Both the High Dysregulation (HD) and Low Dysregulation (LD) groups watched 18 validated video-clips divided in 6 different emotional categories (Erotic, Scenery, Neutral, Sadness, Compassion and Fear) while EEG from 64 electrodes and heart rate (HR) were recorded. Focusing on alpha and gamma EEG rhythms, the connectivity within the VAN network and between VAN and other five relevant networks (DAN, DMN, LN, SMN, VN) was computed and then correlated with the heart rate.

**Results:**

Results showed a different pattern of HR-Network-connectivity correlation in the two groups. EEG Gamma band evidenced several effects only in the HD group with significant positive HR-Network-connectivity correlations for most networks during the Sadness and Neutral movies and to a less extent for Scenery clips (all rs ≥ 0.29, *p* < 0.05).

**Discussion:**

The consistent correlation in HD during Sadness clips points to the relevance of this emotion as a synchronizing agent coordinating cardiovascular and central cortical responses. Unlike the HD, the LD group showed, in the alpha EEG band only, a negative HR-Brain-connectivity correlation in three networks during the Erotic clips, a result that highlights a normal increased attention (bradycardic response) towards relevant biological appetitive cues, while the HD group had an opposite pattern with positive HR- Brain correlation to Erotic in the gamma band that could be explained by greater sexual issues and embarrassment to these stimuli in HD individuals.

## 1 Introduction

The relationship between heart and brain has intrigued not only poets and artists across centuries, but also researchers for the past two decades, with even a specific branch of medicine taking the name of neurocardiology (van der Wall, 2011). In the field of psychophysiology, Thayer and Lane have long proposed the existence of a neurovisceral integration model (Thayer and Lane, 2000), derived from the seminal work of Claude Bernard in the late 1800s (Bernard, 1867). From an anatomical and functional point of view, this model is based on the activity of structures such as the Central Autonomic Network (CAN), a key part of an internal regulatory system that allows the brain to manage visceromotor, neuroendocrine, and behavioral responses that are essential for goal-oriented behavior and adaptability (Benarroch, 1993). The structural bases of the CAN include the anterior cingulate, insular, and ventromedial prefrontal cortices, the central nucleus of the amygdala, the paraventricular and related nuclei of the hypothalamus, the periaqueductal gray matter, the parabrachial nucleus, the nucleus of the solitary tract (NTS), the nucleus ambiguus, the ventrolateral medulla, the ventromedial medulla, and the medullary tegmental field. Its primary output is mediated through the preganglionic sympathetic and parasympathetic neurons, which in turn innervate the heart via the stellate ganglia and the vagus nerve. Consequently, the output of the CAN is directly linked to Heart Rate Variability (HRV). Another important characteristic of this integrated system is the fact that sensory information from organs such as the heart are connected back to the CAN in a feedback loop, for example via the baroreceptor reflex, making communication between heart and brain not only top-down, but also bottom-up. In addition, the authors assert that this model may be crucial in explaining the relationship between cognitive processes and emotional ones, such as self-regulation, especially in the context of disorders in which emotional control is hindered (Thayer and Lane, 2000). More in detail, Cattaneo et al. (2021) recently published a review in which they listed findings pertaining to the relationship between heart rate variability, emotion dysregulation and executive control. Additional support for this comes from studies that found that people suffering from disorders such as Borderline Personality Disorder (BPD) exhibit lower resting vagal tone as compared with healthy controls (Ramesh et al., 2023; Koenig et al., 2016). Notably, these findings on the clinical correlates of low HRV were also confirmed in individuals with high levels of impulsivity and suffering from psychiatric disorders in general (Appelhans and Luecken, 2006; Thayer et al., 2012; Pinna and Edwards, 2020). More generally, the literature on large samples of psychiatric patients showed that most severe disorders exhibit at rest higher Heart Rates (HR, indexing greater distress vulnerability) compared with healthy controls (Latvala et al., 2016; Critchley et al., 2019).

Emotion dysregulation is a complex, multidimensional construct that has often been considered a transdiagnostic marker of disordered psychological functioning (Beauchaine et al., 2007). Concerning the psychophysiology of emotion dysregulation, the vast majority of studies in literature takes into account what can be considered the most representative personality disorder centered around emotion dysregulation as the main key symptom, which is BPD (American Psychiatric Association, 2013). Specifically, studies have reported a decreased activation in prefrontal regions in patients with BPD, both during emotional processing (Schulze et al., 2019) and when they were explicitly instructed to reduce their responses to emotion-triggering stimuli (Schulze et al., 2011). Additionally, individuals with BPD have demonstrated impaired activation of the ventrolateral and dorsolateral prefrontal cortices (vlPFC and dlPFC) when trying to use psychological distancing as a strategy for regulating negative emotions (Koenigsberg et al., 2009). Other research indicated that the dorsal anterior cingulate cortex (dACC) is deactivated during reappraisal tasks in BPD patients (Koenigsberg et al., 2009; Schulze et al., 2011; Lang et al., 2012; van Zutphen et al., 2018). These findings support the notion that, in BPD, difficulties returning to a baseline state following emotional stimuli can be at least partly due to an inadequate regulation from inhibitory brain regions (Neacsiu et al., 2015). When considering the psychophysiological characterization of emotion dysregulation as a subclinical feature, to the best of our knowledge the literature is almost absent. We had previously addressed this literature-gap in a 2022 paper (Fusina et al., 2022) in which we adopted an innovative approach based on source-localized resting state EEG data, to perform a seed-based functional connectivity analysis, in two groups of young women, one with high (HD) and one with low (LD) emotion dysregulation traits. In particular, we focused on Ventral Attention Network (VAN) connectivity due to its relevance in the orienting of attention towards salient stimuli, especially those concerning strong emotional content. The VAN includes the right inferior frontal gyrus (rIFG) and the right temporo-parietal junction (rTPJ) (Samogin et al., 2020), it is the cortical portion of a larger network including deeper structures that comprise the right insula and the right medial temporal regions which include hippocampus and the amygdala. For this reason the VAN is related to an increased elaboration/attention of biologically-emotionally salient stimuli (ElShafei et al., 2020) and their integration with information from the internal state of viscera (interoception) (Fusina et al., 2022). We analyzed activity in the Alpha (8–13 Hz) and Gamma (30–50 Hz) bands and found higher levels of inter- and intra-network VAN connectivity in the high as compared with the low dysregulation group. In particular, we found that Gamma band power in the VAN yielded a positive correlation with measures of emotion dysregulation in the HD group only (Fusina et al., 2022). This represented the first step aimed at analyzing the psychophysiology of emotional dysregulation at a trait level, to better characterize its profile while avoiding the confounding factors commonly found in clinical samples, particularly those related to pharmacological treatment.

Notably, the possibility of performing brain functional connectivity analyses at the source level, has opened the way for investigating brain function in a novel fashion (Marino and Mantini, 2024), by combining an excellent temporal resolution and rich spectral content with a relatively good spatial resolution. Indeed, EEG offers a direct measurement of neural activity with high temporal resolution (in milliseconds) by capturing scalp potentials in real time (Ahmad et al., 2015). EEG is easy to use, silent, cost-effective, and portable, providing high flexibility for various experimental designs and conditions (Glover, 2011). Thus, EEG enhances ecological validity of research, as participants typically are recorded in a comfortable posture, that is, sitting upright in front of a computer screen during EEG recordings (e.g., Spironelli and Angrilli, 2017).

To probe the brain-heart interaction from a psychophysiological perspective, many studies have focused on the relationship between heart rate variability and various neural measures. For example, EEG-derived measures, such as band power time series (Pardo-Rodriguez et al., 2021), power spectral density (Attar et al., 2021), and coherence (Alba et al., 2019) were employed to investigate the brain-heart axis and its relationship with behavior. This has also extended to brain functional connectivity analysis, by means of fMRI (Chang et al., 2013; Colenbier et al., 2022), to identify how heart rate variability was linked to the fluctuations of resting activity in specific brain regions. Other studies also focused on heart rate instead of heart rate variability (Umeno et al., 2003). Indeed, Carr and colleagues pointed out in their review that the use of many HR-related measures could be a potential confound when interpreting findings regarding HRV alterations in clinical populations such as BPD patients (Carr et al., 2018), thus heart rate measured at rest might represent a more direct measure of emotional instability and reactivity in psychiatric populations (Latvala et al., 2016; Critchley et al., 2019).

In this framework, the present research focuses on the investigation of the relationship between brain functional connectivity and heart rate in a population of healthy young women with variable traits of emotion dysregulation. Starting from our past study (Fusina et al., 2022) which analysed brain connectivity during resting state in two groups of selected students, here we focused on Alpha and Gamma EEG bands and VAN as a main hub deserving investigation during emotional stimulation. We interpreted the observed greater between and within-VAN connectivity in high dysregulated participants during resting state as due to their greater attention to visceral emotional responses (Fusina et al., 2022). The Gamma band is particularly effective in the measure of functional connectivity during tasks (Jensen et al., 2007; Womelsdorf and Fries, 2007), thus given the importance of HR in the visceral response, we expected the large observed VAN connectivity to be significantly correlated with HR during a variety of emotional ecological stimulation with standardized film clips.

We enrolled a sample of only women for several reasons: first, due to the well known gender differences in emotional responses (Bradley et al., 2001; Bianchin and Angrilli, 2012) we aimed to reduce sample variance in emotional response in order to focus individual differences especially on emotion dysregulation dimension. Second, the disorders characterized primarily by emotion dysregulation symptoms (such as BPD) affect far more frequently women than men (American Psychiatric Association, 2013). Knowing that Heart Rate (HR) itself may represent an index of emotional reactivity when high (Palomba et al., 2000) or emotional pathological coldness when low (Latvala et al., 2015), we expected that high dysregulation individuals would have exhibited high HR correlated with greater VAN intra- and inter-connectivity. To test the hypothesis we administered participants an ecological emotion stimulation task consisting in the viewing of video clips belonging to different emotional categories. Past research investigated brain connectivity by means of film clips classified in 8 specific emotional categories and using an EEG brain connectivity approach based on graph analysis (Aydın, 2023; Kılıç and Aydın, 2022). This started new innovative research lines investigating the effects of emotions on functional brain connectivity. In the present study we further added to current limited research by investigating the interaction between brain and heart during emotional stimulation. We hypothesized that Gamma band connectivity would positively correlate with heart rate in the high dysregulation group, especially to emotional and high arousal clips. Most importantly, we performed this investigation using state-of-the-art EEG tools to estimate functional connectivity within the brain using a neurophysiological, rather than a neuroimaging, technique. In this study, therefore, we aimed to expand our knowledge on the psychophysiology of emotion dysregulation by focusing not only on the neural features, but also on their relationship with heart-related metrics. In particular, we extracted seed-based functional connectivity values from source-reconstructed EEG data (Samogin et al., 2019) and we assessed the brain-heart interaction in the same population of healthy women with different levels of trait emotion dysregulation that was considered in our previous research (Fusina et al., 2022). Seed-based functional connectivity was used starting from source-reconstructed brain signals. In particular, for each seed, frequency-specific connectivity values were computed by performing a time-frequency decomposition on the neural signals, yielding power envelopes for each frequency. The correlation of band-limited power envelopes, with correction for zero-lag effects (i.e., signal orthogonalization) represented the extracted connectivity metric (Taberna et al., 2024). In this context, we used this approach to assess the correlation between heart rate and brain functional connectivity, with special interest in the Ventral Attention Network activity in the Gamma band with the Alpha band used as a control measure.

## 2 Materials and methods

### 2.1 Participants

Participant selection for our study was conducted by screening an initial pool of 422 female students attending the University of Padova that were recruited via social media. We aimed to obtain two samples, one with high and one with low emotion dysregulation traits. Participants were asked to sign an informed consent form and to fill out three questionnaires that aimed to capture three facets of emotion dysregulation: difficulties in controlling anger, impulsivity and affective lability. A social desirability scale [the Balanced Inventory of Desirable Responding, BIDR-6; Paulhus, 2002; Italian online version by Bobbio and Manganelli (2011)] was also used as a control measure. The questionnaires used for selecting high vs. low dysregulation groups were the following.

The Multidimensional Anger Inventory (MAI; Siegel, 1986) was used to evaluate anger and the difficulties associated with managing it. This inventory consists of 38 items and employs a 5-point Likert scale for responses. Independent experts conducted the translation of the items into Italian, which was subsequently back-translated by a native speaker. The Affective Lability Scales-18 [ALS-18; Oliver and Simons, 2004; Italian version by Contardi et al. (2018)] is an 18-item questionnaire designed to evaluate mood instability. It is a condensed version of the 58-item Affective Lability Scales (Harvey et al., 1989), which measures fluctuations in mood between euthymia and four emotional states: depression, elation, anxiety, and anger. Respondents rate items using a 4-point Likert scale. It has been shown to have a moderate correlation with the Difficulties in Emotion Regulation Scale (DERS; Gratz and Roemer, 2004), with r ≥ 0.4 (Contardi et al., 2018). The UPPS-P Impulsive Behavior Scale [Lynam et al., 2006; Italian version by Fossati et al. (2016)] assesses impulsivity through 59 items that capture different aspects of impulsive behavior by means of a 4-point Likert scale. The Italian version has demonstrated equivalence to other impulsivity assessment tools, such as the Barratt Impulsiveness Scale-11 (BIS-11; Patton et al., 1995). Furthermore, the UPPS-P scales have shown consistent associations with SCID-II (Structured Clinical Interview for DSM- Personality Disorder) BPD symptoms (Fossati et al., 2016).

To obtain a single score, we performed Principal Component Analysis on the data and selected 25 participants scoring above the 85th percentile and 25 participants scoring below the 15th percentile on the first component. The two groups were balanced for age (M- Mean; SD- Standard Deviation, HD-High Dysregulation, LD- Low Dysregulation) (M_HD_ = 22.64, SD_HD_ ± 2.12; M_LD_ = 22.60, SD_LD_ ± 1.63; t_(48)_ = 0.07, n.s.) and lifestyle habits such as coffee intake, smoking, and alcohol use. For further details on questionnaires and sample characteristics, please refer to Fusina et al. (2022). Mean and SD scores for the questionnaires are listed in Table 1.

**Table 1:** Mean age and scores obtained by participants at the questionnaires used for sample selection, with the respective standard deviations (SD).

Group sample age/Questionnaire	High dysregulation group scores *(mean ± SD)*	Low dysregulation group scores *(mean ± SD)*
Age	22.64 ± 2.12	22.60 ± 1.63
ALS-18	35.84 ± 6.76	7.96 ± 5.16
MAI	145.48 ± 10.61	84.04 ± 8.83
UPPS-P	148.32 ± 24.61	114.92 ± 13.12

ALS-18: Affective Lability Scales-18; MAI, Multidimensional Anger Inventory; UPPS-P Impulsive Behavior Scale.

The study was conducted according to the guidelines of the Declaration of Helsinki and approved by the Ethics Committee of Psychology Area, University of Padova (protocol code 2989, date of approval 19/04/2019).

### 2.2 Stimuli

The stimuli that we presented consisted of 18 video clips, each lasting about 2 min (± 10 s) and all had their original soundtrack in Italian language, average sound level was kept constant across all emotional categories, only Scenery clips had a music score while the others had a mix of music and speech as the original film. The clips were divided into six emotional categories according to the emotional content that was presented: Erotic, Scenery, Neutral, Sadness, Compassion and Fear (3 clips per category; see Supplementary Table 1). The stimuli we used were selected among those validated by Maffei and colleagues (Maffei et al., 2015) from the E-MOVIE database (Maffei and Angrilli, 2019), and they were presented in a fixed, pseudo-random order to avoid two clips belonging to the same category being presented consecutively. Soon after clip presentation the participant had to rate some emotional scales included by default in our experiments with E-Movie stimuli (Maffei and Angrilli, 2019). They were: self-perceived Arousal and emotional Valence (9-point Likert scale), and 5 emotional adjectives (5-point Likert Scale, 0–4), that were Embarrassed, Enthusiastic, Inspired, Jittery and Anxious. The only scale that showed a significant Category by Group interaction in which the two groups differed for their response to specific emotional categories was “Embarassed”. For this reason, we included and discussed only the scale on embarrassment. Concerning emotional categories: *Erotic* clips (high valence, high arousal) featured non-pornographic depictions of sexual intercourse between individuals of opposite sexes, serving as a potent and biologically significant stimulus; *Scenery* clips (high valence, low arousal) showcased stunning landscapes accompanied by a sweeping musical score designed to evoke a pleasant and reflective mood in the viewer; *Neutral* clips were sourced from urban documentaries that highlighted various cities around the globe; *Sadness* clips (low valence, low arousal) portrayed characters experiencing feelings of isolation, anguish, distress, and hopelessness, without explicitly showing tears; *Compassion* clips (low valence, medium arousal) depicted characters in tears, often due to loss or separation, aiming to elicit an empathetic response linked to prosocial behavior; *Fear* clips (low valence, high arousal) illustrated individuals facing threats from others, without graphic violence or blood to prevent inducing feelings of disgust.

The selected clips were edited as a continuous stream using Adobe Premiere CS5 and were presented in Full HD resolution (1280×720 pixels) on a 22-inch screen (16:9 aspect ratio). The original source material for the stimuli is illustrated in Supplementary Table 1.

### 2.3 EEG data collection

The experiment was carried out in a dedicated EEG laboratory. EEG data were recorded at 500 Hz sampling rate using an EEG elastic cap (ElectroCap) with 57 tin electrodes on a cap using a SynAmps amplifier (NeuroScan Labs, Sterling, USA). Seven additional external tin electrodes (on nasion, i.e., Nz, left and right external canthi, F9 and F10, below left and right eyes, Ve1 and Ve2, plus the two mastoids) were used for a total of 64 EEG sites. Bandwidth of the amplifier was set to 0–100 Hz (100 Hz low-pass antialiasing filter), 24 bit corresponding to 0.01 uV resolution. All channels were online referred to Cz, later reconstructed by means of re-referencing to average reference. EEG data were acquired during the vision of the short videos described in Paragraph 2.2. During the EEG acquisition, participants were asked to sit back and relax with her eyes open, the arms and legs uncrossed with the feet firmly placed on the floor and arms on the armrests of the chair. They were also asked to look in front of them, and focus on the presented short videos, without visually exploring the room. EEG activity was then recorded continuously from each participant for the duration of all the short movies (around 35 min). HR and EEG analyses were carried out on the entire segment (2 min) of videoclip presentation.

### 2.4 EEG data processing and analysis

An innovative EEG processing pipeline was used to preprocess EEG data (Liu et al., 2017; Marino et al., 2019) and perform seed-based connectivity analysis in the source space (Samogin et al., 2019, 2020). This workflow is highly automated and includes different processing steps, such as EEG signal preprocessing, head model reconstruction, source localization of the preprocessed EEG signal, and connectivity analysis.

Preprocessing includes bad channel detection and artifact removal (Liu et al., 2017; Marino et al., 2019). Bad channel detection was performed based on the values of two parameters: (i) minimum Pearson correlation between each channel signal and all signals from the other channels in the 1–50 Hz frequency range, and (ii) noise variance in the 200–250 Hz frequency range, in which brain activity is considered negligible. Bad channels were considered those in which at least one of these two parameters was an outlier (average value + 4*SD; Liu et al., 2017) compared to the total distribution; the signal from the bad channels was reconstructed by interpolation of the neighboring channels using the FieldTrip toolbox (Oostenveld et al., 2011). Artifact removal was performed following band-pass filtering of the EEG data in the 1–50 Hz band, using EEGLab (Delorme and Makeig, 2004). Independent component analysis was used to separate artifactual contributions from neural activity (ICA; Mantini et al., 2008), and identify the independent components (ICs) linked to eye movement and muscular activity artifacts. A fast fixed-point ICA algorithm (Oja and Yuan, 2006) was used, and artifactual ICs were automatically identified using the procedure described by Liu and colleagues (Liu et al., 2017), based on at least one of the following parameters: (a) the correlation of the IC power with the power of the vertical EOG, and horizontal EOG signals; (b) the IC power spectrum fit against a 1/f function; and (c) the kurtosis of the IC time-course. The thresholds used for each parameter were set in accordance with previous studies (Mantini et al., 2009; De Pasquale et al., 2010; Liu et al., 2017).

The head model reconstruction step is required to project the EEG data in the source space, i.e., to describe the relationship between the recorded EEG data at the scalp level and the neuronal activity generated at the cortical level. This requires an anatomical MR image and the information about the electrode positions. As individual anatomical MR image and electrode positions were not available, the realistic head model was built based on an MRI template and template electrode positions provided by the EEG system manufacturer. The MRI template was segmented in 12 compartments, including brain and cerebellar gray and white matter, brainstem, cerebrospinal fluid, spongy bone, compact bone, muscles, fat, eyeballs and skin. The conductivity values of each compartment were assigned based on previous literature (Haueisen et al., 1997), and a 3D regular 6 mm grid, which overlapped brain (both cortical and subcortical) and cerebellar gray matter compartments, was created to define the location of all possible dipole sources.

The source localization step was performed using the exact low-resolution brain electromagnetic tomography algorithm (eLORETA; Pascual-Marqui et al., 2002).

Starting from the source localized EEG data, the connectivity analysis step was oriented to identify the relationship between the signals from a set of seeds, or region of interests (ROIs), representative of commonly investigated RSNs, including the Default Mode Network (DMN), Dorsal Attention Network (DAN), VAN, Language Network (LN), Somatomotor Network (SMN), and Visual Network (VN). Their coordinates were based on previous research (Samogin et al., 2020). After reconstructing source-space activity in the gray matter, we performed, for each time-course, a time-frequency decomposition with the short-time Fourier transform. Specifically, we used a Hamming window of 2 s, with 50% overlap between consecutive windows, to reconstruct frequencies in the range 1-50 Hz, at steps of 1 Hz. The connectivity value for a given brain rhythm was obtained by averaging the z-value connectivity values calculated for each individual frequency within the relevant range (Alpha, 8–13 Hz; Gamma, 30–50 Hz).

For each ROI, the power spectrum was computed in the range (1–50 Hz). Then, the global power spectrum of each RSN was reconstructed by averaging those constituting its ROIs. The power spectra were analysed in the Alpha (8–13 Hz) and the Gamma (30–50 Hz) frequency bands. Connectivity measurements were achieved by computing Pearson correlations on the logarithmic-transformed signal-orthogonalized power envelopes and then transformed into z-values using the Fisher transform (De Pasquale et al., 2012; Hipp et al., 2012; Samogin et al., 2019). This seed-based approach replicates, using source-localized EEG data, the typical analysis performed using fMRI data, which consists in the definition of specific ROIs from which representative time-courses are extracted and correlated with each other. Hence, the connectivity values represent the correlation values between different pairs of ROIs. When considering the correlation values between the signals from multiple ROIs, belonging to one specific network, we can obtain a single connectivity value by calculating the average of these correlation values. This is defined as intra-network connectivity for that specific network. When considering the correlation values between the signals from multiple ROIs, belonging to two different networks, we define the inter-network connectivity. The EEG connectivity analysis focused on the VAN that previous research showed to be relevant for emotional dysregulation. In particular, intra-network connectivity (VAN) was computed as the average connectivity values between the nodes of the VAN, and inter-network connectivity was computed as the average connectivity between all possible pairs of ROIs belonging to the VAN and any of the other considered networks, i.e., VAN-DAN, VAN-DMN, VAN-LN, VAN-SMN, VAN-VN.

### 2.5 Cardiac signal data collection and analysis

The raw ECG signal was recorded by using two Ag/AgCl cup electrodes, one placed below the left clavicular fossa and one placed at the level of the fifth right intercostal space, in a modified proximal Lead-II configuration. For amplifier settings, see Paragraph 2.3. The ECG signal for each movie clip was exported in Matlab and then processed by means of the Kubios HRV 2.2 software (Tarvainen et al., 2014). Before HR computation, the signal for each clip was visually inspected for artefacts and ectopic heartbeat detection.

### 2.6 Statistical analyses

On the connectivity matrices, Welch’s *t*-tests (Welch, 1947) were computed to assess differences regarding the average connectivity between different networks in the HD group compared with the LD group. To probe the existence of a relationship between HR and VAN connectivity, Spearman’s correlation coefficients were computed between the physiological data and both the intra- and average inter-network connectivity values of the VAN, in each frequency band and for the low and high dysregulation group, separately. The significance level of the correlations was set to *p* < 0.05 (uncorrected) and to p_FDR_ < 0.05 (FDR corrected). False Discovery Rate was employed to correct for multiple comparisons. Also, we reported the connectivity values computer for each condition, for each group, separately for each frequency band.

Concerning heart rate data, to reduce data dimensionality we first computed the average HR per category for each subject. A linear mixed-effects model was then computed, with Category and Group as predictors. A random intercept was included for each subject to account for repeated measures. Linear mixed-effects (LME) models offer a flexible and advantageous statistical approach compared to traditional ANOVA, particularly for repeated measures designs (Krueger and Tian, 2004; Muth et al., 2016). The key benefit of LMEs is their ability to incorporate both fixed effects—variables actively manipulated or controlled—and random effects, which represent factors not directly manipulated but still influencing the data, such as individual differences in within-subject studies (Boisgontier and Cheval, 2016). Additionally, LMEs enable the handling of missing data without resorting to listwise deletion or imputation, unlike ANOVA (Muth et al., 2016).

On the fitted LME models, we further conducted F-tests using the Satterthwaite approximation for degrees of freedom (Luke, 2016) to evaluate the significance of each predictor. Multiple comparisons were again corrected by means of the False Discovery Rate.

## 3 Results

### 3.1 Subjective data

**Figure 1:**
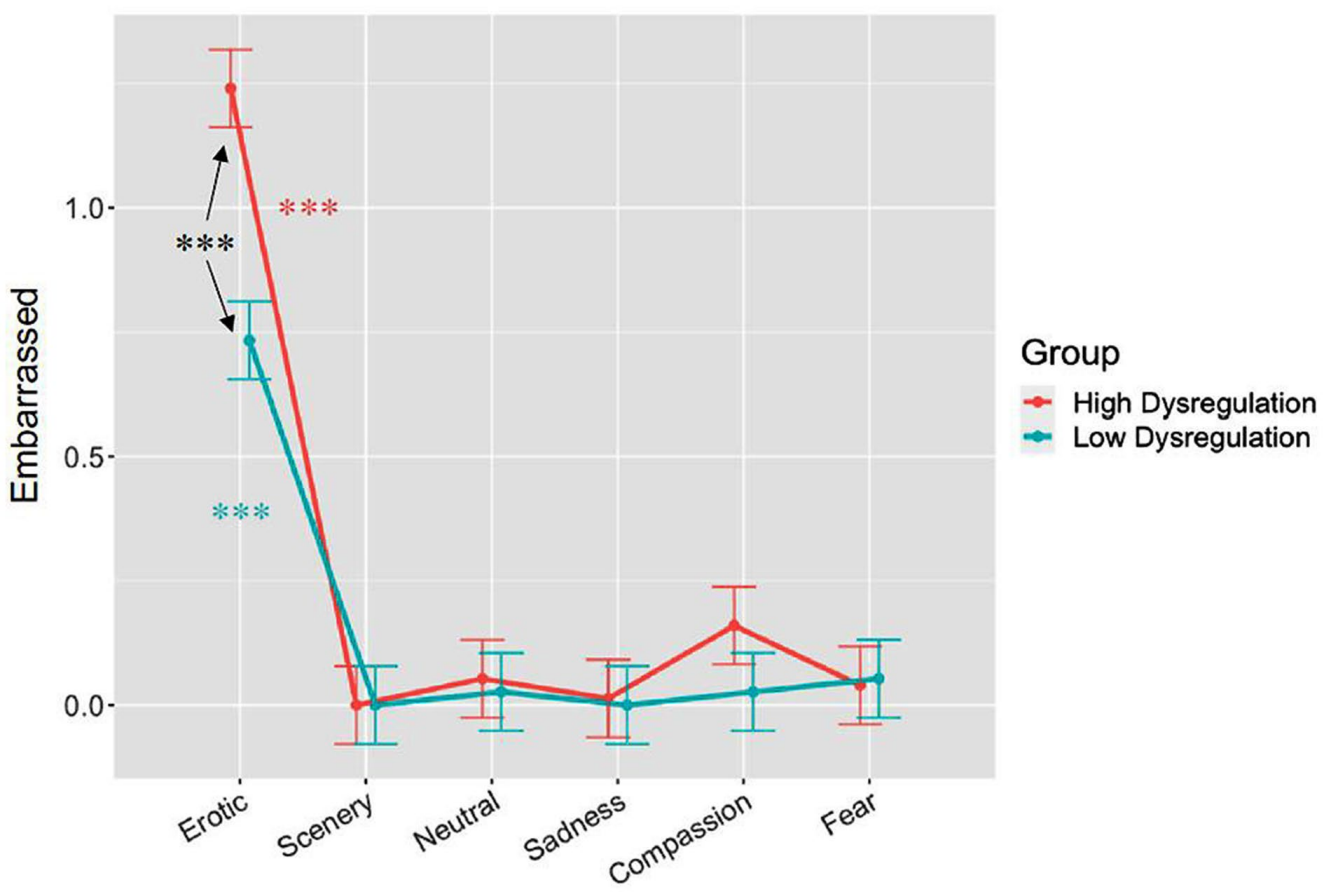
Results of self-evaluated perceived emotional embarrassment on a 4-point analogue scale across the 6 different emotional categories in the two groups. Bars represent SE, asterisks indicate significant effects at *p* < 0.001.

Among all the adjectives describing the subjects’ emotional state, only Embarrassed showed a significant Category by Group interaction. The F-test conducted on the linear mixed-effects model for Embarrassed yielded Category (*F*_(5,240)_ = 50.83, *p* < 0.001) and Group main effects (*F*_(1,48)_ = 5.42, *p* < 0.05), and also a Category*Group interaction (*F*_(5,240)_ = 3.38, *p* < 0.01; Figure 1). Erotic clips were associated with the highest levels of embarrassment, both in the High Dysregulation and in the Low Dysregulation group (all p_FDR_ < 0.001); additionally, the HD group reported feeling more embarrassed than the LD group while viewing Erotic clips (p_FDR_ < 0.001).

### 3.2 Heart rate data

**Figure 2:**
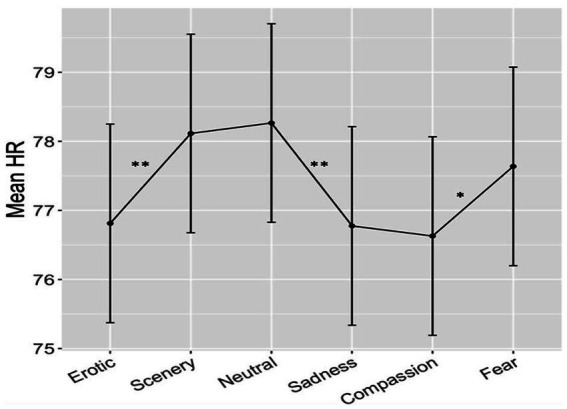
Results of heart rate responses measured in beats-per-minute across the 6 different emotional categories in the two groups. Bars represent SE, asterisks indicate significant effects at *p* < 0.01 (two asterisks) or *p* < 0.05 (one asterisk).

We did not find any significant differences between the two groups both forthe main Group effect and for the Group x Category interaction The linear mixed-effects model on heart rate data, with Category and Group as a predictors, yielded a significant effect of Category only (*F*_(5,220)_ = 5.63, p < 0.001). *Post hoc* analysis revealed significant contrasts between Erotic vs. Scenery, Erotic vs. Neutral, Scenery vs. Sadness, Scenery vs. Compassion, Neutral vs. Sadness, Neutral vs. Compassion, all p_FDR_ < 0.01; and Compassion vs. Fear, pFDR< 0.05 (see Figure 2; bars represent the standard error). More in detail, as shown in Figure 2, Erotic and Sadness clips were associated with lower mean HR than Scenery and Neutral clips, while Compassion clips were associated with lower mean HR than Scenery, Neutral and Fear clips (see Table 2 for details of post-hoc effects).

**Table 2:** Significant post-hoc effects among the 6 emotional conditions in Heart Rate responses (see also Figure 1).

Emotional category	Erotic	Scenery	Neutral	Sadness	Compassion	Fear
Erotic	–					
Scenery	*p* < 0.01	–				
Neutral	*p* < 0.01	n.s.	–			
Sadness	n.s.	*p* < 0.01	*p* < 0.01	–		
Compassion	n.s.	*p* < 0.01	*p* < 0.01	n.s.	–	
Fear	n.s.	n.s.	n.s.	n.s.	*p* < 0.05	–

Ps are FDR corrected.

No effect of Group or of the interaction between Group and Category was found. Nevertheless, the HD group showed a weak overall greater HR (M = 78.28 bpm; SD ± 10.24) with respect to the LD group (M = 76.61 bpm; SD ± 8.41) during all clips. The difference was, on average, 1.67 bpm higher, which is a weak effect (Cohen’s d: 0.178) with respect to the large inter-individual variance, but spread among all clip categories.

### 3.3 EEG and connectivity

**Figure 3:**
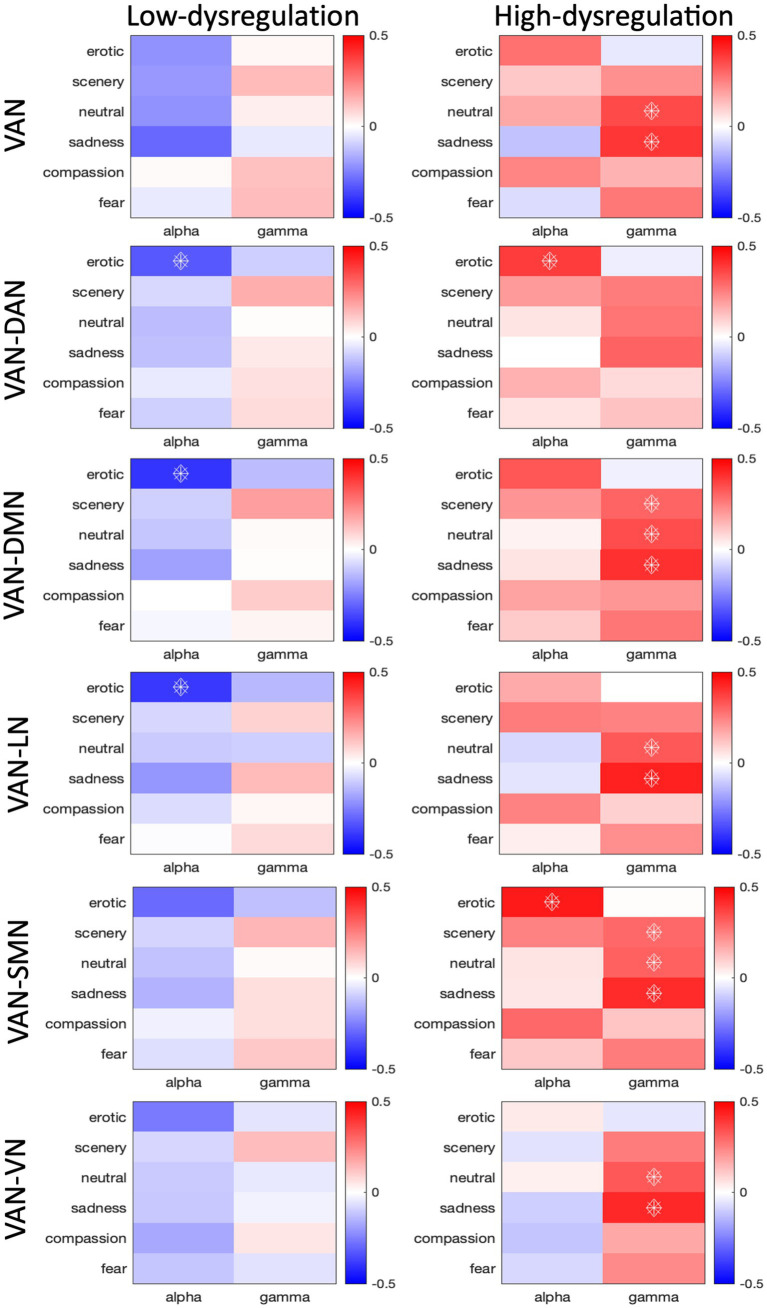
Correlation values between HR and connectivity of intra-VAN and inter-VAN (i.e., VAN-DAN, VAN-DMN, VAN-LN, VAN-SMN, VAN-VN) nodes. The analysis was conducted on low and high dysregulation groups separately. Spearman’s correlation coefficients were calculated for the Alpha and Gamma bands. Correlations with pFDR<0.05 are indicated with diamonds.

Concerning the relationship between HR and VAN connectivity, in the HD group, we found an overall positive relationship (p_FDR_ < 0.05) between both intra- and inter-network VAN connectivity (Supplementary Table 2) and HR in the Gamma band, for all networks apart from the DAN, for the Scenery (VAN-DMN: *r* = 0.30, p_FDR_ < 0.035; VAN-SMN: *r* = 0.29, p_FDR_ < 0.042), Neutral (intra-VAN: *r* = 0.35, p_FDR_ < 0.014; VAN-DMN: *r* = 0.35, p_FDR_ < 0.015; VAN-LN: *r* = 0.33, p_FDR_ < 0.028; VAN-VN: r = 0.33, p_FDR_ < 0.027) and Sadness (intra-VAN: *r* = 0.39, p_FDR_ < 0.010; VAN-DMN: *r* = 0.40, p_FDR_ < 0.007; VAN-LN: *r* = 0.44, p_FDR_ < 0.002; VAN-VN: *r* = 0.42, p_FDR_ < 0.004) categories (Figure 3). Also, significant positive correlations were found in the Alpha band, for VAN-DAN (*r* = 0.30, p_FDR_ < 0.035), and for VAN-SMN (*r* = 0.45, p_FDR_ < 0.002) connections during Erotic clips.

In the LD group, we found an overall negative relationship between VAN-DAN (*r* = −0.32, p_FDR_ < 0.032), VAN-DMN (*r* = −0.40, p_FDR_ < 0.003), and VAN-LN (*r* = −0.39, p_FDR_ < 0.004) connectivity and HR in the Alpha band, for the Erotic category. The differences between the correlation slopes were significant in the comparison between the LD and HD groups in the Alpha band for the Erotic category for both intra- and inter-VAN connectivity (intra-VAN: *z* = −2.864; VAN-DAN: *z* = −4.212; VAN-DMN: *z* = −4.364; VAN-LN: *z* = −3.316; VAN-SMN: *z* = −4.456), for all networks apart from the VN (VAN-VN: *z* = −2.712), for which the most relevant difference in the correlation slope was only in the Gamma band for the Sadness Category (Figure 3).

## 4 Discussion

The objective of the present study was to evaluate the relationship between heart rate and brain functional connectivity, with a particular focus on the Ventral Attention Network (VAN) in the Gamma band, during an ecological emotion stimulation task in two groups of women with high and low levels of emotion dysregulation. The VAN plays a key role in the automatic redirection of attention towards emotional stimuli, which can ultimately disrupt top-down control that would require the individual to focus on different tasks (Iaria et al., 2008; Frank and Sabatinelli, 2012). Our previous findings (Fusina et al., 2022) point to the fact that an intensified focus on internal emotional experiences, particularly when they are negative, may be associated with heightened connectivity between the VAN and other resting-state networks (RSNs). This is particularly important for individuals who struggle with effectively regulating their emotional responses. Indeed, Gamma activity in the VAN has been found to increase during bottom-up attentional control in response to distracting stimuli (ElShafei et al., 2020), and similar patterns have been observed in the primate brain when attention is captured by external stimuli (Buschman and Miller, 2007). Additionally, the Gamma band has been shown to facilitate communication among task-relevant neural nodes (Jensen et al., 2007; Womelsdorf and Fries, 2007). We hypothesized that this increased connectivity in HD, given the greater attention oriented towards their own visceral responses, would have been linked/correlated with cardiovascular reactivity to emotional clips. HR showed an overall emotion-dependent pattern in both groups, with lower HR to Erotic, Sadness and Compassion compared with Scenery, Neutral and Fear. The lower HR during Erotic compared to Neutral clips is well known in the literature on emotions as it marks an increased attention (stimulus intake bradycardia) to biologically salient stimuli (Bianchin and Angrilli, 2012), similarly holds for Sadness and Compassion. The relatively greater HR to Fear compared to the other moderately unpleasant stimuli can be interpreted as due to defense parasympathetic response typically characterized by HR relative acceleration. While the two groups did not differ in this pattern, the HD group nevertheless exhibited overall greater HR with respect to the LD group (+1.67 Bpm). This effect was weak as measured by Cohen’s d (d = 0.18) because of the small sample size, but it is in line with the literature on larger samples, showing that most psychiatric disorders exhibit higher heart rates (indexing greater distress/cardiovascular reactivity) compared with healthy controls (Latvala et al., 2016; Critchley et al., 2019). Taking into account that our HD sample had high traits of emotion dysregulation, with significant greater symptoms of anxiety and depression, the HR found in Emotion Unstable/BPD and Mixed Anxiety/Depression patients by Critchley et al., (2019) was of about the same extent (+1.69 bpm). Thus, this increased HR is a cardiovascular measure that is weakly but significantly associated with psychopathology.

We hypothesized that Gamma band connectivity would have shown a positive correlation with heart rate in the high dysregulation group: namely, greater VAN intra- and inter-connectivity was expected to be correlated with higher HR. This hypothesis was supported both by findings from our prior study (Fusina et al., 2022), in which we found positive correlations between Gamma band activity in the VAN and measures of emotion dysregulation, and by the literature suggesting a co-occurring increase in both Gamma EEG activity and HR during stressful situations, such as, for example, mental arithmetic tasks, with Gamma EEG increase predicting autonomic activation (Umeno et al., 2003). Since we found greater Gamma band VAN connectivity at rest in the HD group as compared to the LD group, and we attributed this to the HD group being generally more focused on their internal emotional experience (Fusina et al., 2022), we expected to replicate these findings also during an ecological emotion stimulation task. In line with our hypothesis, we found a significant positive correlation of Gamma connectivity within the VAN with HR that was higher in the HD group compared with the LD group, especially during the exposure to low-arousal emotions such as those induced in Scenery, Neutral and Sadness clips. In these participants, HR increase also correlated with VAN connectivity of the other resting-state networks, suggesting a global recruitment of attention resources towards their own visceral emotional responses, and this process involved also different brain areas typically not involved in attentive processes. It is noteworthy that the highest correlations between Gamma connectivity in the VAN and HR was found for clips depicting Sadness. This correlation, in addition to being the strongest, was also found when analyzing the interaction between the VAN and all the other networks considered (except for the DAN). Indeed, while it is true that Sadness clips were characterized by low arousal levels, they may also have been especially touched the HD group characterized by greater significant depressive and anxious symptoms compared with the LD group (Fusina et al., 2022). These clips show individuals being alone in scenes of desolation; loneliness has been shown to predict social anxiety, with emotion dysregulation mediating the effect (Eres et al., 2023), and emotion dysregulation levels have been found to partially mediate the longitudinal relationship between loneliness and both depression and stress (Velotti et al., 2021). While for Neutral and Scenery clips, which may have been perceived as more boring – thus possibly triggering a heightened focus on a subjective emotional experience – the positive correlation between Gamma band connectivity in the VAN and HR may retrace the same mechanism that we found during resting state. This suggests that for Sadness clips a different pathway may be involved, but additional studies will be needed to further probe this possibility. The fact that a high HR-Gamma connectivity correlation was not found to other highly arousing unpleasant clips, such as the Fear ones, might be related to low variance in the data for this condition, to physiological response saturation to the most activating emotional stimuli or to the possibility that Sadness (implying social isolation, helplessness, abandonment) represents the most sensitive and salient emotional context triggering emotional worrying in HD women. We think the main agent of this effect is the specificity of sadness clips for HD women. It is also possible that, since in women compared to men, for extreme unpleasant stimuli such as fear, arousal ratings show a ceiling compression effect and a reduction of inter-individual differences (Bradley et al., 2001), this might have reduced both the variance and the size of the correlations.

Concerning the EEG Alpha band, which we considered to represent the control condition, the only significant correlation between HR and VAN connectivity in the LD group was found for Erotic movies, and was in the opposite direction with respect to HD group: while the correlation HR-connectivity in LD was significantly negative (VAN, VAN-LN, VAN-DMN, VAN-DAN) in the HD was positive (VAN-DAN, VAN-SMN). This means that in the Low Dysregulation group HR decreases were associated to Alpha connectivity increases when considering neural communication between VAN nodes and those belonging to the Dorsal Attention, Default Mode and Language Networks. The interpretation of this result is more straightforward, and suggests an attentive effect represented by HR deceleration, stimulus intake, greater orienting of attention to the highly arousing and appetitive content of Erotic clips in the LD group. Our past work evidenced a clear HR deceleration indexing automatic orienting of attention to Erotic stimuli in healthy women (Bianchin and Angrilli, 2012). Instead, in the HD group, increased connectivity in VAN-DAN and VAN-SMN was correlated with higher HR, indexing cardiovascular/sympathetic activation, which is a results opposite to that observed in the LD group. The literature suggests that emotion dysregulation is related to sexual difficulties and dissatisfaction, which points to the fact that Erotic stimuli in particular may elicit a disrupted response in people with problems in regulating their emotions (Fischer et al., 2022). Indeed, our sample showed an interesting effect in line with the above cited literature, in the evaluation of Erotic movies the level of Embarrassment was significantly higher in the HD compared to the LD group (see Figure 1). These findings are especially relevant when considering possible treatment interventions in emotion dysregulation, such as HR biofeedback or neurofeedback, possibly combined with psycho-educational interventions that may teach affected individualsto effectively manage their emotions both at rest and during everyday life situations, for example by means of imagery trainings. The fact that we used movies as a method to induce emotion, which are widely considered to be among the most efficacious stimuli (Westermann et al., 1996; Romeo et al., 2022), is a strong point in our research because it brings subjects closer to real-life experiences when participating in the study, allowing for a more ecological and true-to-life psychophysiological response to take place.

Notwithstanding the innovative approach of this research both in the stimuli that were presented and in the EEG processing methods we adopted, our research is not without limitations. Firstly, as was also stated in (Fusina et al., 2022), the generated head model relied on standard anatomical and electrode positioning templates, which somewhat limited the accuracy of the source localization. Furthermore, the focus on a female-only sample, while having the advantage of allowing us to study a homogeneous sample which has the additional benefit of being especially relevant for the construct of interest (Fusina et al., 2022), is limiting because it does not allow for our findings to be generalizable to the construct of emotion dysregulation as a whole, but only for emotion dysregulation in this specific population and context. Indeed, it would be interesting to probe the same aspects also in a male-only, or better, mixed population to allow a direct comparison between genders.

Furthermore, since to the extent of our knowledge there is very limited literature on these topics, future research will be crucial both in replicating these findings, and also in expanding our understanding of the complex interplay between heart rate measures and functional brain connectivity, especially when considering emotion dysregulation at a subclinical level. Indeed, the study of transdiagnostic markers of psychopathological vulnerability is of the utmost importance when the aim is to develop a more precise and comprehensive understanding of the interplay between physiological systems and how these, in turn, interact with emotions and their regulation. Such an approach could truly enrich both the scientific validity of the theoretical framework underlying these concepts, and the clinical treatment options that may be offered both to patients and to vulnerable, but still healthy, at-risk populations.

## Data Availability

The original contributions presented in the study are included in the article/Supplementary material, further inquiries can be directed to the corresponding author.
