# Supplementary material for: Heart rate and EEG gamma band connectivity in the ventral attention network during emotional movie stimulation in women with high emotion dysregulation
Source: Front Neurosci. 2025 Jun 25;19:1599349. doi: 10.3389/fnins.2025.1599349 (PMC12239019; doi:10.3389/fnins.2025.1599349)
Supplement: Supplementary file 1 [file Data_Sheet_1.docx]

**SUPPLEMENTARY MATERIAL**

**Supplementary Table 1** - Sources from which the used E-MOVIE (Maffei and Angrilli, 2019) clips were taken, divided by category.

| **Category** | **Title** | **Year** | **Director** |
| --- | --- | --- | --- |
| **Erotic** | Lust | 2007 | Ang Lee |
|  | The Notebook | 2004 | Nick Cassavetes |
|  | Monster’s Ball | 2001 | Marc Foster |
| **Scenery** | Great Barrier Reef | 2006 | BBC Planet Earth |
|  | Fresh Water | 2006 | BBC Planet Earth |
|  | Mountains | 2006 | BBC Planet Earth |
| **Neutral** | Paris City Guide | 1996 | Globe Trekker |
|  | London City Guide | 1996 | Globe Trekker |
|  | New York City Guide | 1996 | Globe Trekker |
| **Sadness** | Million Dollar Baby | 2004 | Clint Eastwood |
|  | The Road | 2009 | John Hillcoat |
|  | K-19 | 2002 | Kathryn Bigelow |
| **Compassion** | Lost | 2006 | Chris Sivertson |
|  | The Pursuit of Happyness | 2006 | Gabriele Muccino |
|  | Armageddon | 1998 | Michael Bay |
| **Fear** | The Sixth Sense | 1999 | M. N.Shyamalan |
|  | The Silence of the Lambs | 1991 | Jonathan Demme |
|  | Gothika | 2003 | Mathieu Kassovitz |

**Supplementary Table 2 - Connectivity data:** For each considered frequency band (alpha and gamma), and for each group (low dysregulation, LD, and high dysregulation, HD, we reported the average connectivity (and standard error) between all possible pairs of ROIs belonging to the VAN and any of the other considered networks, i.e., VAN-DAN, VAN-DMN, VAN-LN, VAN-SMN, VAN-VN. Before computing the average connectivity between networks, we computed the connectivity between each pair of ROIs. To this end, for each pair of ROIs, we removed, frequency-by-frequency, the contribution of coherent zero-lag activity from the two time-courses. Then, we applied a logarithmic transformation to the signal-orthogonalized power time-courses, calculated the Pearson correlation between them, and derived z-values by using the r-to-z Fisher’s transform.

**EEG ALPHA Band**

|  | | LD | | | HD | | | statistics |
| --- | --- | --- | --- | --- | --- | --- | --- | --- |
|  | mean | | SE | mean | | SE |  | |
| VAN  Erotic  Scenery  Neutral  Sadness  Compassion  Fear | \| 0.091 \| \| --- \| \| 0.090 \| \| 0.090 \| \| 0.097 \| \| 0.085 \| \| 0.082 \| | | \| 0.007 \| \| --- \| \| 0.006 \| \| 0.007 \| \| 0.009 \| \| 0.007 \| \| 0.008 \| | \| 0.081 \| \| --- \| \| 0.100 \| \| 0.085 \| \| 0.092 \| \| 0.082 \| \| 0.079 \| | | \| 0.008 \| \| --- \| \| 0.010 \| \| 0.010 \| \| 0.010 \| \| 0.009 \| \| 0.008 \| | \| 0.595 \| \| --- \| \| 0.907 \| \| 0.951 \| \| 0.936 \| \| 0.935 \| \| 0.950 \| | |
| VAN-DAN  Erotic  Scenery  Neutral  Sadness  Compassion  Fear | \| 0.052 \| \| --- \| \| 0.062 \| \| 0.069 \| \| 0.071 \| \| 0.067 \| \| 0.064 \| | | \| 0.005 \| \| --- \| \| 0.006 \| \| 0.005 \| \| 0.006 \| \| 0.005 \| \| 0.006 \| | \| 0.056 \| \| --- \| \| 0.063 \| \| 0.070 \| \| 0.071 \| \| 0.066 \| \| 0.067 \| | | \| 0.005 \| \| --- \| \| 0.006 \| \| 0.006 \| \| 0.005 \| \| 0.005 \| \| 0.006 \| | \| 0.716 \| \| --- \| \| 0.940 \| \| 0.951 \| \| 0.989 \| \| 0.935 \| \| 0.950 \| | |
| VAN-DMN  Erotic  Scenery  Neutral  Sadness  Compassion  Fear | \| 0.065 \| \| --- \| \| 0.075 \| \| 0.080 \| \| 0.082 \| \| 0.076 \| \| 0.079 \| | | \| 0.004 \| \| --- \| \| 0.005 \| \| 0.005 \| \| 0.005 \| \| 0.004 \| \| 0.005 \| | \| 0.071 \| \| --- \| \| 0.078 \| \| 0.081 \| \| 0.088 \| \| 0.077 \| \| 0.078 \| | | \| 0.006 \| \| --- \| \| 0.006 \| \| 0.006 \| \| 0.006 \| \| 0.006 \| \| 0.006 \| | \| 0.595 \| \| --- \| \| 0.907 \| \| 0.951 \| \| 0.936 \| \| 0.935 \| \| 0.950 \| | |
| VAN-LN  Erotic  Scenery  Neutral  Sadness  Compassion  Fear | \| 0.042 \| \| --- \| \| 0.061 \| \| 0.057 \| \| 0.062 \| \| 0.058 \| \| 0.054 \| | | \| 0.006 \| \| --- \| \| 0.006 \| \| 0.006 \| \| 0.005 \| \| 0.005 \| \| 0.005 \| | \| 0.051 \| \| --- \| \| 0.059 \| \| 0.065 \| \| 0.066 \| \| 0.055 \| \| 0.063 \| | | \| 0.004 \| \| --- \| \| 0.005 \| \| 0.005 \| \| 0.006 \| \| 0.005 \| \| 0.005 \| | \| 0.595 \| \| --- \| \| 0.907 \| \| 0.951 \| \| 0.936 \| \| 0.935 \| \| 0.950 \| | |
| VAN-SMN  Erotic  Scenery  Neutral  Sadness  Compassion  Fear | \| 0.050 \| \| --- \| \| 0.059 \| \| 0.062 \| \| 0.065 \| \| 0.061 \| \| 0.056 \| | | \| 0.005 \| \| --- \| \| 0.006 \| \| 0.006 \| \| 0.005 \| \| 0.005 \| \| 0.005 \| | \| 0.057 \| \| --- \| \| 0.062 \| \| 0.062 \| \| 0.067 \| \| 0.059 \| \| 0.059 \| | | \| 0.006 \| \| --- \| \| 0.006 \| \| 0.006 \| \| 0.005 \| \| 0.005 \| \| 0.005 \| | \| 0.595 \| \| --- \| \| 0.907 \| \| 0.951 \| \| 0.936 \| \| 0.935 \| \| 0.950 \| | |
| VAN-VN  Erotic  Scenery  Neutral  Sadness  Compassion  Fear | \| 0.047 \| \| --- \| \| 0.047 \| \| 0.052 \| \| 0.061 \| \| 0.054 \| \| 0.051 \| | | \| 0.004 \| \| --- \| \| 0.004 \| \| 0.004 \| \| 0.004 \| \| 0.004 \| \| 0.005 \| | \| 0.048 \| \| --- \| \| 0.060 \| \| 0.056 \| \| 0.063 \| \| 0.056 \| \| 0.056 \| | | \| 0.004 \| \| --- \| \| 0.005 \| \| 0.005 \| \| 0.006 \| \| 0.005 \| \| 0.005 \| | \| 0.778 \| \| --- \| \| 0.361 \| \| 0.951 \| \| 0.936 \| \| 0.935 \| \| 0.950 \| | |

**EEG GAMMA Band**

|  | | LD | | | HD | | | statistics |
| --- | --- | --- | --- | --- | --- | --- | --- | --- |
|  | mean | | SE | mean | | SE |  | |
| VAN  Erotic  Scenery  Neutral  Sadness  Compassion  Fear | \| 0.072 \| \| --- \| \| 0.095 \| \| 0.117 \| \| 0.085 \| \| 0.081 \| \| 0.081 \| | | \| 0.008 \| \| --- \| \| 0.010 \| \| 0.013 \| \| 0.010 \| \| 0.009 \| \| 0.008 \| | \| 0.079 \| \| --- \| \| 0.117 \| \| 0.097 \| \| 0.094 \| \| 0.082 \| \| 0.087 \| | | \| 0.010 \| \| --- \| \| 0.014 \| \| 0.013 \| \| 0.010 \| \| 0.009 \| \| 0.012 \| | \| 0.680 \| \| --- \| \| 0.360 \| \| 0.395 \| \| 0.766 \| \| 0.934 \| \| 0.965 \| | |
| VAN-DAN  Erotic  Scenery  Neutral  Sadness  Compassion  Fear | \| 0.024 \| \| --- \| \| 0.034 \| \| 0.049 \| \| 0.028 \| \| 0.026 \| \| 0.034 \| | | \| 0.006 \| \| --- \| \| 0.007 \| \| 0.007 \| \| 0.005 \| \| 0.007 \| \| 0.006 \| | \| 0.020 \| \| --- \| \| 0.038 \| \| 0.028 \| \| 0.026 \| \| 0.020 \| \| 0.028 \| | | \| 0.005 \| \| --- \| \| 0.007 \| \| 0.006 \| \| 0.005 \| \| 0.006 \| \| 0.006 \| | \| 0.680 \| \| --- \| \| 0.687 \| \| 0.228 \| \| 0.769 \| \| 0.934 \| \| 0.965 \| | |
| VAN-DMN  Erotic  Scenery  Neutral  Sadness  Compassion  Fear | \| 0.037 \| \| --- \| \| 0.050 \| \| 0.066 \| \| 0.045 \| \| 0.043 \| \| 0.049 \| | | \| 0.006 \| \| --- \| \| 0.007 \| \| 0.009 \| \| 0.006 \| \| 0.007 \| \| 0.007 \| | \| 0.040 \| \| --- \| \| 0.061 \| \| 0.047 \| \| 0.048 \| \| 0.038 \| \| 0.048 \| | | \| 0.006 \| \| --- \| \| 0.008 \| \| 0.008 \| \| 0.006 \| \| 0.007 \| \| 0.008 \| | \| 0.680 \| \| --- \| \| 0.360 \| \| 0.351 \| \| 0.769 \| \| 0.934 \| \| 0.965 \| | |
| VAN-LN  Erotic  Scenery  Neutral  Sadness  Compassion  Fear | \| 0.044 \| \| --- \| \| 0.068 \| \| 0.086 \| \| 0.056 \| \| 0.054 \| \| 0.066 \| | | \| 0.007 \| \| --- \| \| 0.009 \| \| 0.012 \| \| 0.008 \| \| 0.009 \| \| 0.009 \| | \| 0.062 \| \| --- \| \| 0.083 \| \| 0.073 \| \| 0.066 \| \| 0.057 \| \| 0.067 \| | | \| 0.009 \| \| --- \| \| 0.011 \| \| 0.010 \| \| 0.008 \| \| 0.008 \| \| 0.009 \| | \| 0.632 \| \| --- \| \| 0.360 \| \| 0.395 \| \| 0.766 \| \| 0.934 \| \| 0.965 \| | |
| VAN-SMN  Erotic  Scenery  Neutral  Sadness  Compassion  Fear | \| 0.032 \| \| --- \| \| 0.045 \| \| 0.064 \| \| 0.038 \| \| 0.034 \| \| 0.046 \| | | \| 0.006 \| \| --- \| \| 0.008 \| \| 0.009 \| \| 0.006 \| \| 0.008 \| \| 0.008 \| | \| 0.037 \| \| --- \| \| 0.062 \| \| 0.049 \| \| 0.046 \| \| 0.035 \| \| 0.048 \| | | \| 0.007 \| \| --- \| \| 0.009 \| \| 0.009 \| \| 0.007 \| \| 0.007 \| \| 0.009 \| | \| 0.680 \| \| --- \| \| 0.360 \| \| 0.395 \| \| 0.766 \| \| 0.934 \| \| 0.965 \| | |
| VAN-VN  Erotic  Scenery  Neutral  Sadness  Compassion  Fear | \| 0.031 \| \| --- \| \| 0.046 \| \| 0.059 \| \| 0.039 \| \| 0.039 \| \| 0.043 \| | | \| 0.006 \| \| --- \| \| 0.007 \| \| 0.008 \| \| 0.006 \| \| 0.007 \| \| 0.006 \| | \| 0.042 \| \| --- \| \| 0.059 \| \| 0.049 \| \| 0.045 \| \| 0.042 \| \| 0.043 \| | | \| 0.007 \| \| --- \| \| 0.008 \| \| 0.008 \| \| 0.006 \| \| 0.007 \| \| 0.007 \| | \| 0.680 \| \| --- \| \| 0.360 \| \| 0.395 \| \| 0.766 \| \| 0.934 \| \| 0.965 \| | |

**Supplementary Table 3 - HR data.** In the following table, mean HR values per movie category per subject are reported. Information about each subject’s group are also given.

| **Subject ID** | **Group** | **Film Category** | **Mean HR** |
| --- | --- | --- | --- |
| 1 | Low Dysregulation | Erotic | 85.04097392 |
| 1 | Low Dysregulation | Scenery | 89.77034029 |
| 1 | Low Dysregulation | Neutral | 88.18061508 |
| 1 | Low Dysregulation | Sadness | 84.80044518 |
| 1 | Low Dysregulation | Compassion | 84.53860339 |
| 1 | Low Dysregulation | Fear | 87.18103572 |
| 3 | Low Dysregulation | Erotic | 71.84547445 |
| 3 | Low Dysregulation | Scenery | 73.16852984 |
| 3 | Low Dysregulation | Neutral | 75.89295865 |
| 3 | Low Dysregulation | Sadness | 72.35649059 |
| 3 | Low Dysregulation | Compassion | 71.79404202 |
| 3 | Low Dysregulation | Fear | 71.40143236 |
| 5 | Low Dysregulation | Erotic | 75.87548875 |
| 5 | Low Dysregulation | Scenery | 76.82515674 |
| 5 | Low Dysregulation | Neutral | 76.97695675 |
| 5 | Low Dysregulation | Sadness | 77.07271696 |
| 5 | Low Dysregulation | Compassion | 75.21562601 |
| 5 | Low Dysregulation | Fear | 76.98689003 |
| 6 | High Dysregulation | Erotic | 56.09840313 |
| 6 | High Dysregulation | Scenery | 57.45925459 |
| 6 | High Dysregulation | Neutral | 60.60389522 |
| 6 | High Dysregulation | Sadness | 57.33122452 |
| 6 | High Dysregulation | Compassion | 58.59207832 |
| 6 | High Dysregulation | Fear | 58.88703306 |
| 7 | Low Dysregulation | Erotic | 76.89119734 |
| 7 | Low Dysregulation | Scenery | 80.10287112 |
| 7 | Low Dysregulation | Neutral | 78.36773508 |
| 7 | Low Dysregulation | Sadness | 79.83665224 |
| 7 | Low Dysregulation | Compassion | 77.73355842 |
| 7 | Low Dysregulation | Fear | 90.90622147 |
| 8 | Low Dysregulation | Erotic | 88.97049786 |
| 8 | Low Dysregulation | Scenery | 92.32990821 |
| 8 | Low Dysregulation | Neutral | 88.45598833 |
| 8 | Low Dysregulation | Sadness | 89.37040948 |
| 8 | Low Dysregulation | Compassion | 89.21208485 |
| 8 | Low Dysregulation | Fear | 89.7431824 |
| 9 | High Dysregulation | Erotic | 75.42784233 |
| 9 | High Dysregulation | Scenery | 75.51166749 |
| 9 | High Dysregulation | Neutral | 75.89676303 |
| 9 | High Dysregulation | Sadness | 75.04461699 |
| 9 | High Dysregulation | Compassion | 74.90371096 |
| 9 | High Dysregulation | Fear | 74.63763126 |
| 10 | Low Dysregulation | Erotic | 79.69712992 |
| 10 | Low Dysregulation | Scenery | 83.90498349 |
| 10 | Low Dysregulation | Neutral | 83.11844171 |
| 10 | Low Dysregulation | Sadness | 77.99743791 |
| 10 | Low Dysregulation | Compassion | 79.19927716 |
| 10 | Low Dysregulation | Fear | 79.85806009 |
| 11 | Low Dysregulation | Erotic | 83.86913371 |
| 11 | Low Dysregulation | Scenery | 85.07840535 |
| 11 | Low Dysregulation | Neutral | 86.15191661 |
| 11 | Low Dysregulation | Sadness | 88.21324925 |
| 11 | Low Dysregulation | Compassion | 87.52305338 |
| 11 | Low Dysregulation | Fear | 87.54220518 |
| 12 | Low Dysregulation | Erotic | 71.77718995 |
| 12 | Low Dysregulation | Scenery | 77.62677087 |
| 12 | Low Dysregulation | Neutral | 78.80836706 |
| 12 | Low Dysregulation | Sadness | 75.41696204 |
| 12 | Low Dysregulation | Compassion | 77.69289122 |
| 12 | Low Dysregulation | Fear | 75.69066292 |
| 13 | High Dysregulation | Erotic | 67.46435472 |
| 13 | High Dysregulation | Scenery | 67.66880499 |
| 13 | High Dysregulation | Neutral | 68.49895214 |
| 13 | High Dysregulation | Sadness | 67.12315713 |
| 13 | High Dysregulation | Compassion | 65.83248123 |
| 13 | High Dysregulation | Fear | 66.26467425 |
| 14 | Low Dysregulation | Erotic | 68.04205645 |
| 14 | Low Dysregulation | Scenery | 70.7283428 |
| 14 | Low Dysregulation | Neutral | 72.67906345 |
| 14 | Low Dysregulation | Sadness | 68.16259114 |
| 14 | Low Dysregulation | Compassion | 68.65866044 |
| 14 | Low Dysregulation | Fear | 67.92574073 |
| 15 | High Dysregulation | Erotic | 90.50161603 |
| 15 | High Dysregulation | Scenery | 95.06324706 |
| 15 | High Dysregulation | Neutral | 94.23818674 |
| 15 | High Dysregulation | Sadness | 92.12947492 |
| 15 | High Dysregulation | Compassion | 89.42808115 |
| 15 | High Dysregulation | Fear | 95.89695054 |
| 16 | High Dysregulation | Erotic | 60.31089961 |
| 16 | High Dysregulation | Scenery | 64.65316578 |
| 16 | High Dysregulation | Neutral | 66.49592959 |
| 16 | High Dysregulation | Sadness | 63.71027518 |
| 16 | High Dysregulation | Compassion | 63.82997557 |
| 16 | High Dysregulation | Fear | 65.65117131 |
| 17 | Low Dysregulation | Erotic | 71.07182095 |
| 17 | Low Dysregulation | Scenery | 71.19227798 |
| 17 | Low Dysregulation | Neutral | 73.12258683 |
| 17 | Low Dysregulation | Sadness | 71.93037941 |
| 17 | Low Dysregulation | Compassion | 70.74803515 |
| 17 | Low Dysregulation | Fear | 76.75635926 |
| 18 | High Dysregulation | Erotic | 93.48436163 |
| 18 | High Dysregulation | Scenery | 93.25896982 |
| 18 | High Dysregulation | Neutral | 93.07366254 |
| 18 | High Dysregulation | Sadness | 88.83610242 |
| 18 | High Dysregulation | Compassion | 88.58829429 |
| 18 | High Dysregulation | Fear | 93.86805943 |
| 19 | Low Dysregulation | Erotic | 74.91037543 |
| 19 | Low Dysregulation | Scenery | 74.33953203 |
| 19 | Low Dysregulation | Neutral | 72.9105883 |
| 19 | Low Dysregulation | Sadness | 72.10902798 |
| 19 | Low Dysregulation | Compassion | 69.63823605 |
| 19 | Low Dysregulation | Fear | 70.04967725 |
| 20 | High Dysregulation | Erotic | 63.46686538 |
| 20 | High Dysregulation | Scenery | 60.54452109 |
| 20 | High Dysregulation | Neutral | 63.84556707 |
| 20 | High Dysregulation | Sadness | 61.7569375 |
| 20 | High Dysregulation | Compassion | 61.96875103 |
| 20 | High Dysregulation | Fear | 61.53028755 |
| 21 | Low Dysregulation | Erotic | 85.05082784 |
| 21 | Low Dysregulation | Scenery | 90.34966359 |
| 21 | Low Dysregulation | Neutral | 91.62441424 |
| 21 | Low Dysregulation | Sadness | 88.95203992 |
| 21 | Low Dysregulation | Compassion | 88.21113526 |
| 21 | Low Dysregulation | Fear | 88.87084788 |
| 22 | High Dysregulation | Erotic | 79.44774583 |
| 22 | High Dysregulation | Scenery | 67.31445192 |
| 22 | High Dysregulation | Neutral | 68.23457559 |
| 22 | High Dysregulation | Sadness | 67.58244532 |
| 22 | High Dysregulation | Compassion | 67.51678853 |
| 22 | High Dysregulation | Fear | 68.07314844 |
| 23 | Low Dysregulation | Erotic | 76.25540143 |
| 23 | Low Dysregulation | Scenery | 80.78094609 |
| 23 | Low Dysregulation | Neutral | 81.46586675 |
| 23 | Low Dysregulation | Sadness | 79.92755189 |
| 23 | Low Dysregulation | Compassion | 77.46424824 |
| 23 | Low Dysregulation | Fear | 77.82695098 |
| 24 | High Dysregulation | Erotic | 80.58501553 |
| 24 | High Dysregulation | Scenery | 84.97688457 |
| 24 | High Dysregulation | Neutral | 82.21730968 |
| 24 | High Dysregulation | Sadness | 82.31443364 |
| 24 | High Dysregulation | Compassion | 81.09073834 |
| 24 | High Dysregulation | Fear | 82.99312723 |
| 25 | High Dysregulation | Erotic | 85.83274808 |
| 25 | High Dysregulation | Scenery | 83.22984488 |
| 25 | High Dysregulation | Neutral | 88.98213589 |
| 25 | High Dysregulation | Sadness | 85.99906846 |
| 25 | High Dysregulation | Compassion | 87.64550587 |
| 25 | High Dysregulation | Fear | 88.3684497 |
| 26 | Low Dysregulation | Erotic | 75.06928676 |
| 26 | Low Dysregulation | Scenery | 77.39774318 |
| 26 | Low Dysregulation | Neutral | 79.43114044 |
| 26 | Low Dysregulation | Sadness | 75.77036304 |
| 26 | Low Dysregulation | Compassion | 76.62992752 |
| 26 | Low Dysregulation | Fear | 76.4821757 |
| 27 | Low Dysregulation | Erotic | 68.29220167 |
| 27 | Low Dysregulation | Scenery | 68.68770488 |
| 27 | Low Dysregulation | Neutral | 69.4315976 |
| 27 | Low Dysregulation | Sadness | 68.16993381 |
| 27 | Low Dysregulation | Compassion | 71.01815459 |
| 27 | Low Dysregulation | Fear | 69.22980044 |
| 28 | High Dysregulation | Erotic | 87.5555947 |
| 28 | High Dysregulation | Scenery | 88.34615102 |
| 28 | High Dysregulation | Neutral | 88.42216041 |
| 28 | High Dysregulation | Sadness | 85.90292842 |
| 28 | High Dysregulation | Compassion | 86.17146613 |
| 28 | High Dysregulation | Fear | 87.79022587 |
| 29 | High Dysregulation | Erotic | 88.71082195 |
| 29 | High Dysregulation | Scenery | 88.68320151 |
| 29 | High Dysregulation | Neutral | 90.49046851 |
| 29 | High Dysregulation | Sadness | 89.35277655 |
| 29 | High Dysregulation | Compassion | 88.15981872 |
| 29 | High Dysregulation | Fear | 88.72639346 |
| 30 | Low Dysregulation | Erotic | 86.23917635 |
| 30 | Low Dysregulation | Scenery | 85.54352821 |
| 30 | Low Dysregulation | Neutral | 86.38534271 |
| 30 | Low Dysregulation | Sadness | 85.73750877 |
| 30 | Low Dysregulation | Compassion | 85.38644047 |
| 30 | Low Dysregulation | Fear | 83.98683914 |
| 31 | High Dysregulation | Erotic | 80.44253822 |
| 31 | High Dysregulation | Scenery | 82.77472127 |
| 31 | High Dysregulation | Neutral | 80.83116764 |
| 31 | High Dysregulation | Sadness | 81.69905871 |
| 31 | High Dysregulation | Compassion | 80.98624227 |
| 31 | High Dysregulation | Fear | 81.42324116 |
| 32 | Low Dysregulation | Erotic | 58.86068342 |
| 32 | Low Dysregulation | Scenery | 62.18597301 |
| 32 | Low Dysregulation | Neutral | 59.62537343 |
| 32 | Low Dysregulation | Sadness | 61.17334922 |
| 32 | Low Dysregulation | Compassion | 60.7285081 |
| 32 | Low Dysregulation | Fear | 62.04630963 |
| 34 | Low Dysregulation | Erotic | 78.07973181 |
| 34 | Low Dysregulation | Scenery | 79.08170267 |
| 34 | Low Dysregulation | Neutral | 79.05375311 |
| 34 | Low Dysregulation | Sadness | 75.2577976 |
| 34 | Low Dysregulation | Compassion | 76.83670789 |
| 34 | Low Dysregulation | Fear | 75.60315844 |
| 35 | High Dysregulation | Erotic | 84.38723725 |
| 35 | High Dysregulation | Scenery | 93.33055471 |
| 35 | High Dysregulation | Neutral | 90.10671901 |
| 35 | High Dysregulation | Sadness | 91.22316652 |
| 35 | High Dysregulation | Compassion | 89.21378242 |
| 35 | High Dysregulation | Fear | 89.38291115 |
| 36 | Low Dysregulation | Erotic | 76.83253277 |
| 36 | Low Dysregulation | Scenery | 76.04439324 |
| 36 | Low Dysregulation | Neutral | 78.59383475 |
| 36 | Low Dysregulation | Sadness | 76.38544528 |
| 36 | Low Dysregulation | Compassion | 75.85148024 |
| 36 | Low Dysregulation | Fear | 76.62065538 |
| 37 | High Dysregulation | Erotic | 72.71650501 |
| 37 | High Dysregulation | Scenery | 76.74264983 |
| 37 | High Dysregulation | Neutral | 76.42672422 |
| 37 | High Dysregulation | Sadness | 75.22961369 |
| 37 | High Dysregulation | Compassion | 76.5051868 |
| 37 | High Dysregulation | Fear | 75.05586425 |
| 39 | Low Dysregulation | Erotic | 60.84784168 |
| 39 | Low Dysregulation | Scenery | 62.8262342 |
| 39 | Low Dysregulation | Neutral | 63.12767582 |
| 39 | Low Dysregulation | Sadness | 59.70920745 |
| 39 | Low Dysregulation | Compassion | 60.65310683 |
| 39 | Low Dysregulation | Fear | 61.56273347 |
| 40 | High Dysregulation | Erotic | 72.96892263 |
| 40 | High Dysregulation | Scenery | 74.86852773 |
| 40 | High Dysregulation | Neutral | 72.84689905 |
| 40 | High Dysregulation | Sadness | 72.15357505 |
| 40 | High Dysregulation | Compassion | 72.21222179 |
| 40 | High Dysregulation | Fear | 71.8414585 |
| 41 | Low Dysregulation | Erotic | 73.48899529 |
| 41 | Low Dysregulation | Scenery | 74.77955064 |
| 41 | Low Dysregulation | Neutral | 71.97319709 |
| 41 | Low Dysregulation | Sadness | 70.58084078 |
| 41 | Low Dysregulation | Compassion | 71.12594746 |
| 41 | Low Dysregulation | Fear | 71.87684895 |
| 42 | Low Dysregulation | Erotic | 75.16991025 |
| 42 | Low Dysregulation | Scenery | 70.69317538 |
| 42 | Low Dysregulation | Neutral | 74.39421051 |
| 42 | Low Dysregulation | Sadness | 72.10230982 |
| 42 | Low Dysregulation | Compassion | 72.84072353 |
| 42 | Low Dysregulation | Fear | 70.16768438 |
| 43 | Low Dysregulation | Erotic | 72.02071713 |
| 43 | Low Dysregulation | Scenery | 72.7292936 |
| 43 | Low Dysregulation | Neutral | 67.22443572 |
| 43 | Low Dysregulation | Sadness | 71.66506698 |
| 43 | Low Dysregulation | Compassion | 71.16465641 |
| 43 | Low Dysregulation | Fear | 78.97135264 |
| 44 | High Dysregulation | Erotic | 81.66482061 |
| 44 | High Dysregulation | Scenery | 83.55894953 |
| 44 | High Dysregulation | Neutral | 85.2087644 |
| 44 | High Dysregulation | Sadness | 81.6886012 |
| 44 | High Dysregulation | Compassion | 83.24514646 |
| 44 | High Dysregulation | Fear | 81.63740658 |
| 45 | Low Dysregulation | Erotic | 74.27270547 |
| 45 | Low Dysregulation | Scenery | 77.55363945 |
| 45 | Low Dysregulation | Neutral | 77.71460489 |
| 45 | Low Dysregulation | Sadness | 74.63274292 |
| 45 | Low Dysregulation | Compassion | 74.58620995 |
| 45 | Low Dysregulation | Fear | 75.62674764 |
| 46 | High Dysregulation | Erotic | 62.11553328 |
| 46 | High Dysregulation | Scenery | 63.18607137 |
| 46 | High Dysregulation | Neutral | 61.79673116 |
| 46 | High Dysregulation | Sadness | 61.65754162 |
| 46 | High Dysregulation | Compassion | 63.28236542 |
| 46 | High Dysregulation | Fear | 61.20677524 |
| 47 | Low Dysregulation | Erotic | 89.84031539 |
| 47 | Low Dysregulation | Scenery | 78.71522703 |
| 47 | Low Dysregulation | Neutral | 77.88582611 |
| 47 | Low Dysregulation | Sadness | 79.98901141 |
| 47 | Low Dysregulation | Compassion | 79.23879747 |
| 47 | Low Dysregulation | Fear | 87.13521791 |
| 48 | High Dysregulation | Erotic | 70.8387149 |
| 48 | High Dysregulation | Scenery | 72.66864311 |
| 48 | High Dysregulation | Neutral | 70.26742515 |
| 48 | High Dysregulation | Sadness | 70.40566627 |
| 48 | High Dysregulation | Compassion | 71.06531518 |
| 48 | High Dysregulation | Fear | 71.24268939 |
| 49 | High Dysregulation | Erotic | 82.03128596 |
| 49 | High Dysregulation | Scenery | 85.55862285 |
| 49 | High Dysregulation | Neutral | 84.94312111 |
| 49 | High Dysregulation | Sadness | 82.54071991 |
| 49 | High Dysregulation | Compassion | 82.42187518 |
| 49 | High Dysregulation | Fear | 78.64063247 |
| 50 | High Dysregulation | Erotic | 95.4821362 |
| 50 | High Dysregulation | Scenery | 98.1441065 |
| 50 | High Dysregulation | Neutral | 100.3428241 |
| 50 | High Dysregulation | Sadness | 97.13284946 |
| 50 | High Dysregulation | Compassion | 95.07170783 |
| 50 | High Dysregulation | Fear | 96.40958283 |

**Supplementary Table 4 - Embarrassment data.** In the following table, mean Embarrassment values per movie category per subject are reported. Information about each subject’s group is also given.

| **Subject ID** | **Group** | **Film Category** | **Embarrassed** |
| --- | --- | --- | --- |
| 1 | Low Dysregulation | Erotic | 0.666666667 |
| 1 | Low Dysregulation | Scenery | 0 |
| 1 | Low Dysregulation | Neutral | 0 |
| 1 | Low Dysregulation | Sadness | 0 |
| 1 | Low Dysregulation | Compassion | 0 |
| 1 | Low Dysregulation | Fear | 0 |
| 2 | High Dysregulation | Erotic | 0 |
| 2 | High Dysregulation | Scenery | 0 |
| 2 | High Dysregulation | Neutral | 0 |
| 2 | High Dysregulation | Sadness | 0 |
| 2 | High Dysregulation | Compassion | 1 |
| 2 | High Dysregulation | Fear | 0.333333333 |
| 3 | Low Dysregulation | Erotic | 0 |
| 3 | Low Dysregulation | Scenery | 0 |
| 3 | Low Dysregulation | Neutral | 0 |
| 3 | Low Dysregulation | Sadness | 0 |
| 3 | Low Dysregulation | Compassion | 0 |
| 3 | Low Dysregulation | Fear | 0 |
| 4 | High Dysregulation | Erotic | 1 |
| 4 | High Dysregulation | Scenery | 0 |
| 4 | High Dysregulation | Neutral | 0 |
| 4 | High Dysregulation | Sadness | 0 |
| 4 | High Dysregulation | Compassion | 0 |
| 4 | High Dysregulation | Fear | 0 |
| 5 | Low Dysregulation | Erotic | 0.666666667 |
| 5 | Low Dysregulation | Scenery | 0 |
| 5 | Low Dysregulation | Neutral | 0 |
| 5 | Low Dysregulation | Sadness | 0 |
| 5 | Low Dysregulation | Compassion | 0 |
| 5 | Low Dysregulation | Fear | 0 |
| 6 | High Dysregulation | Erotic | 2.666666667 |
| 6 | High Dysregulation | Scenery | 0 |
| 6 | High Dysregulation | Neutral | 0 |
| 6 | High Dysregulation | Sadness | 0 |
| 6 | High Dysregulation | Compassion | 0 |
| 6 | High Dysregulation | Fear | 0 |
| 7 | Low Dysregulation | Erotic | 2.333333333 |
| 7 | Low Dysregulation | Scenery | 0 |
| 7 | Low Dysregulation | Neutral | 0 |
| 7 | Low Dysregulation | Sadness | 0 |
| 7 | Low Dysregulation | Compassion | 0.666666667 |
| 7 | Low Dysregulation | Fear | 0 |
| 8 | Low Dysregulation | Erotic | 1.333333333 |
| 8 | Low Dysregulation | Scenery | 0 |
| 8 | Low Dysregulation | Neutral | 0 |
| 8 | Low Dysregulation | Sadness | 0 |
| 8 | Low Dysregulation | Compassion | 0 |
| 8 | Low Dysregulation | Fear | 0.333333333 |
| 9 | High Dysregulation | Erotic | 1 |
| 9 | High Dysregulation | Scenery | 0 |
| 9 | High Dysregulation | Neutral | 0 |
| 9 | High Dysregulation | Sadness | 0 |
| 9 | High Dysregulation | Compassion | 0 |
| 9 | High Dysregulation | Fear | 0 |
| 10 | Low Dysregulation | Erotic | 1 |
| 10 | Low Dysregulation | Scenery | 0 |
| 10 | Low Dysregulation | Neutral | 0 |
| 10 | Low Dysregulation | Sadness | 0 |
| 10 | Low Dysregulation | Compassion | 0 |
| 10 | Low Dysregulation | Fear | 0 |
| 11 | Low Dysregulation | Erotic | 1 |
| 11 | Low Dysregulation | Scenery | 0 |
| 11 | Low Dysregulation | Neutral | 0 |
| 11 | Low Dysregulation | Sadness | 0 |
| 11 | Low Dysregulation | Compassion | 0 |
| 11 | Low Dysregulation | Fear | 0 |
| 12 | Low Dysregulation | Erotic | 0 |
| 12 | Low Dysregulation | Scenery | 0 |
| 12 | Low Dysregulation | Neutral | 0 |
| 12 | Low Dysregulation | Sadness | 0 |
| 12 | Low Dysregulation | Compassion | 0 |
| 12 | Low Dysregulation | Fear | 0 |
| 13 | High Dysregulation | Erotic | 0.333333333 |
| 13 | High Dysregulation | Scenery | 0 |
| 13 | High Dysregulation | Neutral | 0.666666667 |
| 13 | High Dysregulation | Sadness | 0 |
| 13 | High Dysregulation | Compassion | 1 |
| 13 | High Dysregulation | Fear | 0 |
| 14 | Low Dysregulation | Erotic | 1.333333333 |
| 14 | Low Dysregulation | Scenery | 0 |
| 14 | Low Dysregulation | Neutral | 0 |
| 14 | Low Dysregulation | Sadness | 0 |
| 14 | Low Dysregulation | Compassion | 0 |
| 14 | Low Dysregulation | Fear | 0.666666667 |
| 15 | High Dysregulation | Erotic | 2.333333333 |
| 15 | High Dysregulation | Scenery | 0 |
| 15 | High Dysregulation | Neutral | 0 |
| 15 | High Dysregulation | Sadness | 0 |
| 15 | High Dysregulation | Compassion | 0 |
| 15 | High Dysregulation | Fear | 0 |
| 16 | High Dysregulation | Erotic | 1.666666667 |
| 16 | High Dysregulation | Scenery | 0 |
| 16 | High Dysregulation | Neutral | 0 |
| 16 | High Dysregulation | Sadness | 0 |
| 16 | High Dysregulation | Compassion | 0 |
| 16 | High Dysregulation | Fear | 0 |
| 17 | Low Dysregulation | Erotic | 1.666666667 |
| 17 | Low Dysregulation | Scenery | 0 |
| 17 | Low Dysregulation | Neutral | 0 |
| 17 | Low Dysregulation | Sadness | 0 |
| 17 | Low Dysregulation | Compassion | 0 |
| 17 | Low Dysregulation | Fear | 0 |
| 18 | High Dysregulation | Erotic | 2.666666667 |
| 18 | High Dysregulation | Scenery | 0 |
| 18 | High Dysregulation | Neutral | 0 |
| 18 | High Dysregulation | Sadness | 0.333333333 |
| 18 | High Dysregulation | Compassion | 0.333333333 |
| 18 | High Dysregulation | Fear | 0.666666667 |
| 19 | Low Dysregulation | Erotic | 2.666666667 |
| 19 | Low Dysregulation | Scenery | 0 |
| 19 | Low Dysregulation | Neutral | 0 |
| 19 | Low Dysregulation | Sadness | 0 |
| 19 | Low Dysregulation | Compassion | 0 |
| 19 | Low Dysregulation | Fear | 0 |
| 20 | High Dysregulation | Erotic | 0 |
| 20 | High Dysregulation | Scenery | 0 |
| 20 | High Dysregulation | Neutral | 0 |
| 20 | High Dysregulation | Sadness | 0 |
| 20 | High Dysregulation | Compassion | 0 |
| 20 | High Dysregulation | Fear | 0 |
| 21 | Low Dysregulation | Erotic | 0.333333333 |
| 21 | Low Dysregulation | Scenery | 0 |
| 21 | Low Dysregulation | Neutral | 0 |
| 21 | Low Dysregulation | Sadness | 0 |
| 21 | Low Dysregulation | Compassion | 0 |
| 21 | Low Dysregulation | Fear | 0 |
| 22 | High Dysregulation | Erotic | 0 |
| 22 | High Dysregulation | Scenery | 0 |
| 22 | High Dysregulation | Neutral | 0 |
| 22 | High Dysregulation | Sadness | 0 |
| 22 | High Dysregulation | Compassion | 0 |
| 22 | High Dysregulation | Fear | 0 |
| 23 | Low Dysregulation | Erotic | 0 |
| 23 | Low Dysregulation | Scenery | 0 |
| 23 | Low Dysregulation | Neutral | 0 |
| 23 | Low Dysregulation | Sadness | 0 |
| 23 | Low Dysregulation | Compassion | 0 |
| 23 | Low Dysregulation | Fear | 0 |
| 24 | High Dysregulation | Erotic | 2.333333333 |
| 24 | High Dysregulation | Scenery | 0 |
| 24 | High Dysregulation | Neutral | 0.333333333 |
| 24 | High Dysregulation | Sadness | 0 |
| 24 | High Dysregulation | Compassion | 0 |
| 24 | High Dysregulation | Fear | 0 |
| 25 | High Dysregulation | Erotic | 1.333333333 |
| 25 | High Dysregulation | Scenery | 0 |
| 25 | High Dysregulation | Neutral | 0 |
| 25 | High Dysregulation | Sadness | 0 |
| 25 | High Dysregulation | Compassion | 0 |
| 25 | High Dysregulation | Fear | 0 |
| 26 | Low Dysregulation | Erotic | 0.333333333 |
| 26 | Low Dysregulation | Scenery | 0 |
| 26 | Low Dysregulation | Neutral | 0 |
| 26 | Low Dysregulation | Sadness | 0 |
| 26 | Low Dysregulation | Compassion | 0 |
| 26 | Low Dysregulation | Fear | 0 |
| 27 | Low Dysregulation | Erotic | 0.666666667 |
| 27 | Low Dysregulation | Scenery | 0 |
| 27 | Low Dysregulation | Neutral | 0 |
| 27 | Low Dysregulation | Sadness | 0 |
| 27 | Low Dysregulation | Compassion | 0 |
| 27 | Low Dysregulation | Fear | 0 |
| 28 | High Dysregulation | Erotic | 1.333333333 |
| 28 | High Dysregulation | Scenery | 0 |
| 28 | High Dysregulation | Neutral | 0 |
| 28 | High Dysregulation | Sadness | 0 |
| 28 | High Dysregulation | Compassion | 0.333333333 |
| 28 | High Dysregulation | Fear | 0 |
| 29 | High Dysregulation | Erotic | 0.666666667 |
| 29 | High Dysregulation | Scenery | 0 |
| 29 | High Dysregulation | Neutral | 0 |
| 29 | High Dysregulation | Sadness | 0 |
| 29 | High Dysregulation | Compassion | 0.333333333 |
| 29 | High Dysregulation | Fear | 0 |
| 30 | Low Dysregulation | Erotic | 0.666666667 |
| 30 | Low Dysregulation | Scenery | 0 |
| 30 | Low Dysregulation | Neutral | 0 |
| 30 | Low Dysregulation | Sadness | 0 |
| 30 | Low Dysregulation | Compassion | 0 |
| 30 | Low Dysregulation | Fear | 0 |
| 31 | High Dysregulation | Erotic | 2.333333333 |
| 31 | High Dysregulation | Scenery | 0 |
| 31 | High Dysregulation | Neutral | 0 |
| 31 | High Dysregulation | Sadness | 0 |
| 31 | High Dysregulation | Compassion | 0.333333333 |
| 31 | High Dysregulation | Fear | 0 |
| 32 | Low Dysregulation | Erotic | 2 |
| 32 | Low Dysregulation | Scenery | 0 |
| 32 | Low Dysregulation | Neutral | 0 |
| 32 | Low Dysregulation | Sadness | 0 |
| 32 | Low Dysregulation | Compassion | 0 |
| 32 | Low Dysregulation | Fear | 0 |
| 33 | High Dysregulation | Erotic | 1 |
| 33 | High Dysregulation | Scenery | 0 |
| 33 | High Dysregulation | Neutral | 0 |
| 33 | High Dysregulation | Sadness | 0 |
| 33 | High Dysregulation | Compassion | 0 |
| 33 | High Dysregulation | Fear | 0 |
| 34 | Low Dysregulation | Erotic | 0.333333333 |
| 34 | Low Dysregulation | Scenery | 0 |
| 34 | Low Dysregulation | Neutral | 0 |
| 34 | Low Dysregulation | Sadness | 0 |
| 34 | Low Dysregulation | Compassion | 0 |
| 34 | Low Dysregulation | Fear | 0 |
| 35 | High Dysregulation | Erotic | 0.333333333 |
| 35 | High Dysregulation | Scenery | 0 |
| 35 | High Dysregulation | Neutral | 0 |
| 35 | High Dysregulation | Sadness | 0 |
| 35 | High Dysregulation | Compassion | 0 |
| 35 | High Dysregulation | Fear | 0 |
| 36 | Low Dysregulation | Erotic | 0 |
| 36 | Low Dysregulation | Scenery | 0 |
| 36 | Low Dysregulation | Neutral | 0 |
| 36 | Low Dysregulation | Sadness | 0 |
| 36 | Low Dysregulation | Compassion | 0 |
| 36 | Low Dysregulation | Fear | 0 |
| 37 | High Dysregulation | Erotic | 2.666666667 |
| 37 | High Dysregulation | Scenery | 0 |
| 37 | High Dysregulation | Neutral | 0.333333333 |
| 37 | High Dysregulation | Sadness | 0 |
| 37 | High Dysregulation | Compassion | 0.333333333 |
| 37 | High Dysregulation | Fear | 0 |
| 38 | High Dysregulation | Erotic | 0.666666667 |
| 38 | High Dysregulation | Scenery | 0 |
| 38 | High Dysregulation | Neutral | 0 |
| 38 | High Dysregulation | Sadness | 0 |
| 38 | High Dysregulation | Compassion | 0 |
| 38 | High Dysregulation | Fear | 0 |
| 39 | Low Dysregulation | Erotic | 0.333333333 |
| 39 | Low Dysregulation | Scenery | 0 |
| 39 | Low Dysregulation | Neutral | 0 |
| 39 | Low Dysregulation | Sadness | 0 |
| 39 | Low Dysregulation | Compassion | 0 |
| 39 | Low Dysregulation | Fear | 0 |
| 40 | High Dysregulation | Erotic | 3.333333333 |
| 40 | High Dysregulation | Scenery | 0 |
| 40 | High Dysregulation | Neutral | 0 |
| 40 | High Dysregulation | Sadness | 0 |
| 40 | High Dysregulation | Compassion | 0 |
| 40 | High Dysregulation | Fear | 0 |
| 41 | Low Dysregulation | Erotic | 0 |
| 41 | Low Dysregulation | Scenery | 0 |
| 41 | Low Dysregulation | Neutral | 0 |
| 41 | Low Dysregulation | Sadness | 0 |
| 41 | Low Dysregulation | Compassion | 0 |
| 41 | Low Dysregulation | Fear | 0 |
| 42 | Low Dysregulation | Erotic | 0 |
| 42 | Low Dysregulation | Scenery | 0 |
| 42 | Low Dysregulation | Neutral | 0 |
| 42 | Low Dysregulation | Sadness | 0 |
| 42 | Low Dysregulation | Compassion | 0 |
| 42 | Low Dysregulation | Fear | 0 |
| 43 | Low Dysregulation | Erotic | 0 |
| 43 | Low Dysregulation | Scenery | 0 |
| 43 | Low Dysregulation | Neutral | 0 |
| 43 | Low Dysregulation | Sadness | 0 |
| 43 | Low Dysregulation | Compassion | 0 |
| 43 | Low Dysregulation | Fear | 0 |
| 44 | High Dysregulation | Erotic | 0.333333333 |
| 44 | High Dysregulation | Scenery | 0 |
| 44 | High Dysregulation | Neutral | 0 |
| 44 | High Dysregulation | Sadness | 0 |
| 44 | High Dysregulation | Compassion | 0 |
| 44 | High Dysregulation | Fear | 0 |
| 45 | Low Dysregulation | Erotic | 0 |
| 45 | Low Dysregulation | Scenery | 0 |
| 45 | Low Dysregulation | Neutral | 0.333333333 |
| 45 | Low Dysregulation | Sadness | 0 |
| 45 | Low Dysregulation | Compassion | 0 |
| 45 | Low Dysregulation | Fear | 0.333333333 |
| 46 | High Dysregulation | Erotic | 0.333333333 |
| 46 | High Dysregulation | Scenery | 0 |
| 46 | High Dysregulation | Neutral | 0 |
| 46 | High Dysregulation | Sadness | 0 |
| 46 | High Dysregulation | Compassion | 0 |
| 46 | High Dysregulation | Fear | 0 |
| 47 | Low Dysregulation | Erotic | 1 |
| 47 | Low Dysregulation | Scenery | 0 |
| 47 | Low Dysregulation | Neutral | 0.333333333 |
| 47 | Low Dysregulation | Sadness | 0 |
| 47 | Low Dysregulation | Compassion | 0 |
| 47 | Low Dysregulation | Fear | 0 |
| 48 | High Dysregulation | Erotic | 1.666666667 |
| 48 | High Dysregulation | Scenery | 0 |
| 48 | High Dysregulation | Neutral | 0 |
| 48 | High Dysregulation | Sadness | 0 |
| 48 | High Dysregulation | Compassion | 0 |
| 48 | High Dysregulation | Fear | 0 |
| 49 | High Dysregulation | Erotic | 0 |
| 49 | High Dysregulation | Scenery | 0 |
| 49 | High Dysregulation | Neutral | 0 |
| 49 | High Dysregulation | Sadness | 0 |
| 49 | High Dysregulation | Compassion | 0 |
| 49 | High Dysregulation | Fear | 0 |
| 50 | High Dysregulation | Erotic | 1 |
| 50 | High Dysregulation | Scenery | 0 |
| 50 | High Dysregulation | Neutral | 0 |
| 50 | High Dysregulation | Sadness | 0 |
| 50 | High Dysregulation | Compassion | 0.333333333 |
| 50 | High Dysregulation | Fear | 0 |
